# Structural differences in gut bacteria communities in developmental stages of natural populations of *Lutzomyia evansi* from Colombia's Caribbean coast

**DOI:** 10.1186/s13071-016-1766-0

**Published:** 2016-09-13

**Authors:** Rafael José Vivero, Natalia Gil Jaramillo, Gloria Cadavid-Restrepo, Sandra I. Uribe Soto, Claudia Ximena Moreno Herrera

**Affiliations:** 1 Grupo de Investigación en Sistemática Molecular, Universidad Nacional de Colombia, Street 59 A # 63-20, Medellín, Postal Code 050003 Colombia; 2 PECET (Programa de Estudio y Control de Enfermedades Tropicales), Universidad de Antioquia, Street 62 # 52-59, SIU-Sede de Investigación Universitaria, Laboratory 632, Medellín, Postal Code 050003 Colombia; 3 Grupo de Microbiodiversidad y Bioprospección, Laboratorio de Biología Celular y Molecular, Universidad Nacional de Colombia sede Medellín, Street 59 A # 63-20, Medellín, Postal Code 050003 Colombia

**Keywords:** Sand flies, Immature, Adults, Vector, Gut, Microbiota

## Abstract

**Background:**

*Lutzomyia evansi*, a phlebotomine insect endemic to Colombia’s Caribbean coast, is considered to be the main vector of visceral and cutaneous leishmaniasis in the region. Although insects of this species can harbor pathogenic and non-pathogenic microorganisms in their intestinal microbiota, there is little information available about the diversity of gut bacteria present in *Lutzomyia evansi*. In this study, conventional microbiological methods and molecular tools were used to assess the composition of bacterial communities associated with *Lutzomyia evansi* guts in immature and adult stages of natural populations from the department of Sucre (Caribbean coast of Colombia).

**Methods:**

Sand flies were collected from two locations (peri-urban and jungle biotype) in the Department of Sucre (Caribbean coast of Colombia). A total of 752 *Lutzomyia evansi* intestines were dissected. In this study, 125 bacterial strains were isolated from different culture media (LB Agar, MacConkey Agar). Different methods were used for bacterial identification, including ribosomal intergenic spacer analysis (RISA) and analysis of the 16S rRNA and *gyr*B gene sequences. The genetic profiles of the bacterial populations were generated and temporal temperature gradient gel electrophoresis (TTGE) was used to compare them with total gut DNA. We also used PCR and DNA sequence analysis to determine the presence of *Wolbachia* endosymbiont bacteria and *Leishmania* parasites.

**Results:**

The culture-dependent technique showed that the dominant intestinal bacteria isolated belong to *Acinetobacter*, *Enterobacter*, *Pseudomonas*, *Ochrobactrum*, *Shinella* and *Paenibacillus* in the larval stage; *Lysobacter*, *Microbacterium*, *Streptomyces*, *Bacillus* and *Rummeliibacillus* in the pupal stage; and *Staphylococcus*, *Streptomyces*, *Brevibacterium*, *Acinetobacter*, *Enterobacter* and *Pantoea* in the adult stage. Statistical analysis revealed significant differences between the fingerprint patterns of the PCR-TTGE bands in bacterial communities from immature and adult stages. Additionally, differences were found in bacterial community structure in fed females, unfed females, males and larvae. The intestinal bacteria detected by PCR-TTGE were *Enterobacter cloacae* and *Bacillus thuringiensis*, which were present in different life stages of *Lu. evansi*, and *Burkholderia cenocepacia* and *Bacillus gibsonii*, which were detected only in the larval stage. *Wolbachia* and *Leishmania* were not detected in gut samples of *Lutzomyia evansi*.

**Conclusions:**

The analyses conducted using microbiological and molecular approaches indicated significant variations in the bacterial communities associated with the gut of *Lu. evansi*, depending on the developmental stage and food source. We propose that these elements affect microbial diversity in *L. evansi* guts and may in turn influence pathogen transmission to humans bitten by this insect.

## Background

Insects from the genus *Lutzomyia* França, 1924 (subfamily Phlebotominae) are of great interest in biological studies because of their ability to transmit parasites of the genus *Leishmania* Ross, 1903 [1] to vertebrates, as well as other pathogens, including the bacterium *Bartonella baciliformis* and viruses from the families *Bunyaviridae*, *Reoviridae* and *Rhabdoviridae* [2–4].

The potential of *Lutzomyia* spp. to transmit parasites can be attributed primarily to the hematophagic habits of females, which are the only ones that ingest blood as a necessary source of protein for the maturation of their eggs [5]. Various studies on feeding preferences suggest that phlebotomine insects have eclectic feeding habits and can take blood from different vertebrates [5, 6]. There are also autogenous species that use protein stocks acquired during the larval stage to complete their first gonadotropic cycle [7]. For survival and movement, sap, nectar or aphid secretions are the most-described carbohydrate sources [8].

As described above, phlebotomine insects need a varied diet and have the ability to digest complex molecules throughout the different stages of their development [9, 10]. The different bacterial communities these insects acquire from the environment and via their eating habits are involved in the plant material digestion process and promote the availability of iron from erythrocytes and essential amino acids and vitamins from the B complex [11, 12].

The metabolic importance of bacterial communities associated with the guts of phlebotomine insects has led to the emergence of several new questions related to the parasite-vector interaction. The most basic and essential of these include what bacterial communities are present in the gut and how they interact with parasites [13]. Other questions relate to the location of bacteria in the intestine and other organs of physiological relevance [13], the identity of bacteria that remain during metamorphosis [14], the ability of bacteria to express recombinant molecules that display antitrypanosomal or antiviral activity [15], and the existence of intestinal bacteria with insecticidal properties or the ability to alter insect life-cycles [16].

In America, bacterial diversity analysis has only been recorded for two species: *Lutzomyia longipalpis* and *Lu. cruzi* (adult stages from colonies and natural populations in Brazil and Colombia) [17–20]. The gut bacteria isolates registered for these species mainly belong to the genera *Serratia*, *Enterobacter*, *Acinetobacter* and *Pseudomonas* [19]. Microbiota associated with immature stages in natural breeding sites have not yet been evaluated, due to several difficulties related to their recovery and identification [21]. The large number of *Lutzomyia* spp. in Colombia serves as a motivation for exploring gut bacteria in species that have been found naturally infected with *Leishmania*.

Around 163 species from the subfamily Phlebotominae are registered in Colombia. Fourteen of these have been reported as vectors for different *Leishmania* spp. [22]; however *Lu. longipalpis* is the only species whose midgut microbiota have been described [19, 20]. *Lutzomyia evansi* is the most abundant on Colombia’s Caribbean coast; this species transmits *Leishmania infantum chagasi* and *Leishmania braziliensis* [23]. Studies have been conducted in order to understand the role of *Lu. evansi* as a vector of *Leishmania infantum* (vector competence, life-cycle, insecticide resistance, genetic structure, blood intake and bionomics) [24]. However, the composition of bacterial communities present in the gut and the changes in the microbiota associated with food sources, developmental stages, and the presence of parasite infections, have not been studied in this species. Among the gut microbiota, molecular detection and identification of endosymbionts such as *Wolbachia* is important due to their potential use for decreasing the population density of *Lutzomyia* species and interfering with parasite multiplication and thus, with *Leishmania* transmission [13, 16, 17].

The several feeding events of natural populations of *Lu. evansi* and their interaction with the environment (including multiple hosts), allows species to acquire a complex and dynamic microbiome. In recent years, a polyphasic approach incorporating classical microbiological techniques and culture-independent methods based on direct analysis of DNA (or RNA) cultures has gained popularity. This approach has had reliable and effective results with regard to detecting and identifying microorganisms, leading to major advances in the understanding of this complex microbial ecosystem [13, 16, 19]. For the culture-independent approach, fingerprinting methods represent a complete alternative for the study of intestinal microbiota. These methods include temperature gradient gel electrophoresis (TTGE), which is a powerful genetic tool for establishing distinctive profiles of microbial communities among different samples [25, 26].

The culture-dependent techniques, allow access to cultivable bacteria that may have anti-leishmanicidal and entomopathogenic activity. It is a platform bioprospecting, for this reason it is necessary to include bacterial isolation as a first phase. TTGE allows a quick screenshot of the abundance and presence of different bacterial communities. Therefore, these two approaches should be complementary.

The aim of our study was to investigate the diversity of bacterial communities associated with the guts of two natural populations (jungle and peri-urban biotype) of *Lu. evansi* in different developmental stages (adult and immature), through culture-dependent and culture-independent methods. This species was abundant in the endemic foci of visceral leishmaniasis on Colombia’s Caribbean coast and representative of the sand fly diversity in this area.

## Methods

### Collection, processing and identification of sand flies

Sand flies were collected from two locations in the Department of Sucre (Caribbean coast of Colombia). The first location was associated with a peri-urban biotype near the Ovejas municipality (75°13'W, 9°31'N; 277 metres above sea level, masl), classified as a tropical dry forest ecosystem [27]. The second location corresponded to a jungle biotype at the “Primates” Wildlife Experimental Station (09°31'48.0"N, 75°21'4.3"W, 220 masl) in the municipality of Colosó, located within the Protected Forest Reserve “Serranía de Coraza”. This nature reserve is classified as a transitional ecosystem of premontane dry forest to tropical dry forest [27]. Adult stage samples were collected from Ovejas and Colosó during July and October 2013 and January 2014, while immature stages were searched for in Ovejas during October and November 2013.

The adult specimens were collected from the two locations using Shannon-type extra-domiciliary white light traps that remained active between 18:00 and 22:00 h. The adult sand flies collected were transported live to the laboratory in entomological cages to obtain the gut and morphological structures (male genitalia and female spermathecae) for taxonomic identification of *Lu. evansi* using a taxonomic key [28].

Immature stages of *Lu. evansi* (larvae and pupae) were isolated from substrates extracted from potential breeding sites, usually at the base of *Cordia dentata* shrubs (common name: Uvito) associated with the urban environment of Ovejas (results not shown). The search was performed with a stereomicroscope, using the direct view technique [21]. Immature specimens were immediately processed to obtain the gut; the rest of each specimen (integument and head) was stored at -20 °C for DNA extraction [29]. The barcode region of the cytochrome *c* oxidase I gene [30] was amplified and high quality sequences were obtained in order to estimate the taxonomic identity of the immature specimens by comparing them with adult specimen sequences from the same locality and others reported in the NCBI GenBank database (results not shown).

### *Lutzomyia evansi* guts

**Fig. 1:**
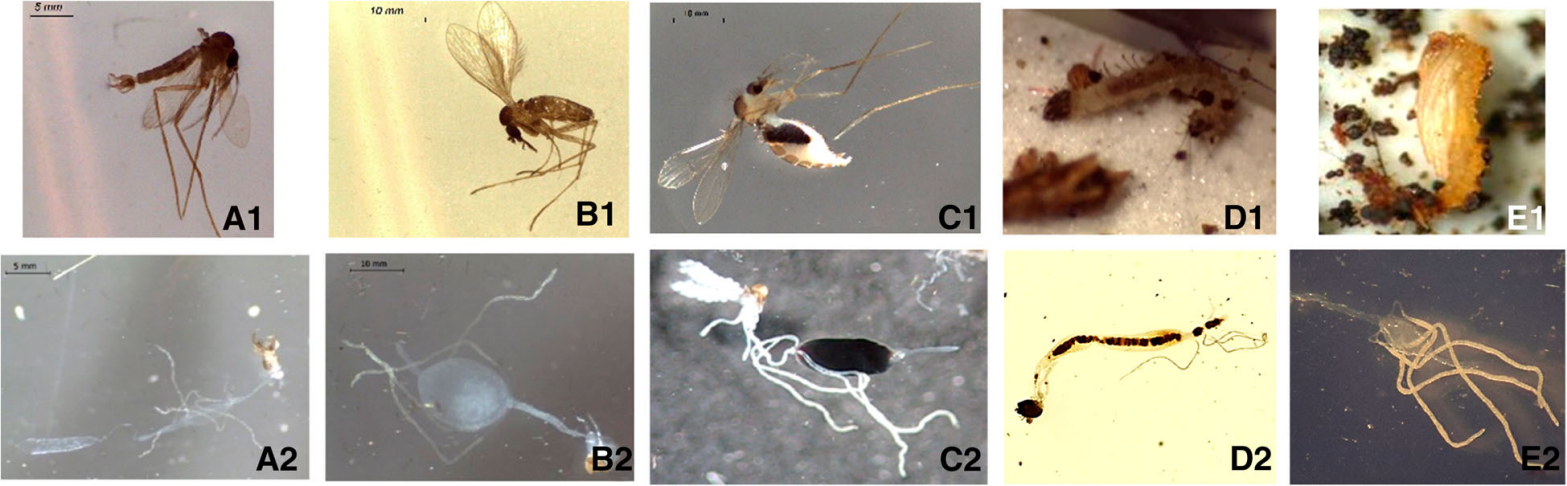
Dissection and obtaining of guts. **A1**: male; **A2**: gut of a male; **B1**: unfed female; **B2**: gut of an unfed female; **C1**: fed female; **C2**: gut of fed female; **D1**: fourth-instar larvae recovered in natural breeding sites; **D2**: gut of a fourth-instar larva recovered in natural breeding sites; **E1**: pupa recovered in natural breeding sites; **E2**: gut of a pupa recovered in natural breeding sites

Processed adult sand flies confirmed as *Lu. evansi* were categorized into three groups and represented by source as follows: fed females (Colosó coded as FFC; Ovejas coded as FFO), unfed females (Colosó coded as UFC; Ovejas coded as UFO) and males (Colosó coded as MC; Ovejas coded as MV) (Fig. 1). Isolation of sand fly guts was conducted in a sterile environment. Prior to gut dissection, adult and immature specimens were washed with 50 μl of 1× PBS (Phosphate Buffered Saline) and Tween 20, centrifuged at 3000× *g* for 5 min, and submerged in a 70 % ethanol wash for 2 min to remove excess microvilli, dust and exogenous bacteria; the latter were removed. After the head was removed, the gut was microdissected and isolated aseptically with stilettos on a sterile glass slide under a stereoscope in 1× PBS buffer. Intestinal homogenate was obtained by lysis of the epithelium, only when the gut was placed in sterile eppendorf tubes, to ensure the absence of external bacteria. This procedure was developed according to previously described protocols on intestinal microbiota sand flies [14, 17, 18, 20]. The rest of the dissected insect body was mounted on a slide for morphological identification.

Gut pools were formed according to locality and physiological stage; these were re-suspended in 100 μl of sterile PBS. Gut groups (*n* = 50) were macerated and then vortexed for 30 s to break the intestinal wall and obtain intestinal homogenate. The guts of larvae (Ovejas, L4) and pupae (Ovejas, PP) (Fig. 1), were processed individually prior to taxonomic identification. Each intestinal homogenate was preserved and processed cold. Half was used for conventional microbiological methods of cultivable fraction (CF) and the remainder was used for the culture-independent molecular approach of the total intestinal bacteria fraction (TF). Given the lack of knowledge on the biology of immature stages and the lack of taxonomic keys for these forms, in this study the number of samples of immature specimens was lower and processed individually with respect to adult stages. To date no article of intestinal microbiota has included immature stages isolated from natural breeding sites.

### Culture-dependent assays

#### Isolation of bacteria and colony-forming units

In this study, cultivable and aerobic midgut bacteria were isolated. We used methods and culture media described in other studies on intestinal microbiota in sand flies. Gut homogenates were cultured using serial dilutions for surface plating on LB Agar (Merck, Bogota, Colombia) and MacConkey Agar (Merck, Bogota, Colombia) [31]. The plates were incubated aerobically at 33 °C for 24 to 48 h, as described in previous studies [14, 17, 18, 20]. LB Agar was chosen as a non-selective medium to promote growth of a diversity of microbes including nutritionally fastidious bacteria. MacConkey agar is a selective and differential culture medium for bacteria designed to selectively isolate Gram-negative and enteric (normally found in the intestinal tract) bacilli and differentiate them based on lactose fermentation. A test tube containing LB (Merck) broth opened near the dissection area constituted our sterility control during the dissection process. The total number of colony-forming units (CFU) was determined for each homogenate. The isolates selected for molecular identification were purified; they were then characterized macroscopically based on colony characteristics and microscopically by Gram staining. All isolates were cryopreserved in 20 % glycerol at -80 °C. Pearson Chi-Square tests based on the colony counting was performed using SPSS software ver. 18 to detect statistical differences in bacterial populations isolated from *Lu. evansi*.

#### *PCR amplification of the internal transcribed spacer* (*ITS*) *between the 23S and 16S ribosomal gene*, *16S rRNA gene and gyr*B *gene*

For total DNA extraction, each colony was incubated for 10 min at 95 °C in 100 μl of sterile Tris-EDTA 1X to generate cell lysis. This was followed by centrifugation at 10,000 rpm for 5 min, in order to obtain the DNA in the supernatant for use as a template in the PCR assays (colony PCR Protocol) [32]. The template DNA of all isolates was initially assessed by amplifying the ITS region using the primers L1 (5'-CAA GGC ATC CAC CGT-3') and G1 (5'-GAA GTC GTA ACA AGG-3') [32] as previously described [33]. GelCompar II software (Applied Maths Biosystems, Belgium) was used to build a dendrogram with the banding patterns of the ITS region. A distance matrix was calculated using the Dice coefficient [34] and cluster analysis was performed using the unweighted arithmetic average algorithm (UPGMA) [35].

In order to properly represent the bacterial diversity associated with guts of adult and immature *Lu. evansi*, similarity of ≥ 70 % between ITS standards was established as a criterion for selecting bacterial isolates to be considered for the subsequent molecular identification assays. The DNA isolated from the selected colonies was used to amplify the 16S rRNA gene (1.5 kbp), using the primers Eubac 27 F (5'-AGA GTT TGA TCC TGG CTC AG-3') and 1492R (5'-GGT TAC CTT GTT ACG ACT T-3' [32] as previously described [36]. The *gyr*B gene (encodes the *B*-subunit of DNA gyrase, a type II DNA topoisomerase) was amplified from the same template DNA of some isolates [37]. The primers UP1 (5'-GAA GTC ATC ATG ACC GTT CTG CAY GCN GGN GGN AAR TTY GA-3') and UP-2r (5'-AGC AGG GTA CGG ATG TGC GAG CCR TCN ACR TCN GCR TCN GTC AT-3') were used with a reaction mixture and a thermal profile designed to amplify two conserved regions of approximately 1.26 kbp of the *gyr*B gene [38]. Positive and negative controls (DNA from pure cultures of *Escherichia coli* or *Bacillus cereus* and ultrapure water) were routinely included in all PCR reactions for both genes.

PCR amplicons of partial 16S rRNA and the *gyr*B gene were verified by visualization in 1 % agarose gels and purified using Wizard PCR Preps (Promega, Medellin, Colombia) and the double-stranded DNA was sequenced in both directions using the ABI PRISM 3700 DNA analyzer service (Applied Biosystems) of Macrogen Company Inc. in Korea. The nucleotide sequences of the 16S rRNA and *gyr*B genes reported in this study were submitted to the NCBI GenBank database (Tables 2 and 4).

#### Bacterial identity and phylogenetic relationships

The sequences of the 16S rRNA and *gyr*B genes were edited using Bioedit v7.2.5 [39], in order to obtain consensus sequences for each isolate. Subsequently, they were compared with GenBank and RDP reference sequences, using BLASTN (National Center for Biotechnology Information; http://blast.ncbi.nlm.nih.gov/Blast.cgi) to confirm the identity and the taxonomic identity of the nucleotide fragment obtained, with ≥ 97 % similarity values. The ClustalW algorithm built in MEGA 5 [40] was used to align sequences of the 16S rRNA and *gyr*B genes with reference sequences of bacterial species found in GenBank as a result of the similarity search. The sequence matrix of delimited fragments of the 16S rRNA and gyrB genes was used to generate neighbor-joining dendrograms [41], using the DNA correction distances of the Kimura 2-parameter model [42] to provide a graphical representation of the clustering patterns between species. Verification and/or validation of recombination events and the presence of chimeras were carried out with RDP4 software [43] to ensure the accuracy of the nucleotide variability with respect to the sequences previously reported in the GenBank database.

### Culture-independent assays

#### Temporal temperature gradient gel electrophoresis (PCR-TTGE)

TTGE protocol was used to monitor the dynamic changes in the intestinal microbe population in adult and immature specimens. Total gut DNA was extracted using the Ultra CleanTM Soil DNA Isolation Kit (MO BIO Laboratories, Inc., California, USA), according to the manufacturer’s instructions. Final DNA aliquots were quantified using an ND-100 Nanodrop Thermo Scientific spectrophotometer (Thermo Fisher Scientific Inc, Massachusetts, USA). The quality of genomic DNA was analyzed on 1 % agarose gels, followed by EZ-visionTM DNA 6X STAIN (AMRESCO). DNA samples were subjected to 16S rRNA gene amplification (V3–V6 variable regions) to obtain a 566 bp fragment with primers specific to the conserved domains. These were 341 F (5'-CCT GCA GGA GGC AGC AG-3'), with an extra GC termination at the 5' end, and 907R (5'-CCC TGA CGT GTT ATT CAA TTC Y-3') [44]. PCR reaction conditions were implemented as described previously [45].

All PCR products were checked for amplification by electrophoresis on 1 % (w/v) agarose gels. PCR products were concentrated with Concentrator (Eppendorf 5301) and dissolved in 20 μl of double-distilled H_2_O. Approximately 600 ng of amplified DNA were loaded into each well. TTGE was performed using the DCode TM Universal mutation detection system (Bio-Rad Laboratories, Hercules, CA, USA) with a 6 % (w/v) polyacrylamide-7 M urea gel and a 1.25× TAE running buffer (40 mM Tris base, 20 mM sodium acetate, 1 mM EDTA) at a constant voltage of 55 V for 15 h. The initial temperature was 66 °C, the final temperature was 69 °C, and a ramp of 0.2 °C h^-1^ was applied. Additionally, each gel contained at least two marker lanes consisting of a 100 bp ladder (Fermentas, Burlington, USA) and another consisting of 16S fragments of two reference bacterial strains (*Enterobacter cloacae*: KU134778 and *Acinetobacter colcaeceticus*: KU134748).

Polyacrylamide gels were stained with SafeView™ DNA stain (Applied Biological Materials, Richmond, Canada) and imaged with a digital scanner. Banding patterns were analyzed through Pearson correlation and Complete Linkage clustering [46] using GelCompar II software (Applied Biosystems Maths, Massachusetts, USA) [47], in order to detect differences in diversity and abundance profiles among the gut samples. Bands of interest were excised and the DNA was eluted in 60 μl of ultrapure water. Five microliters of the DNA elution were re-amplified using the same primers, 341 F (without the GC tail) and 907R. Bands that were successfully re-amplified were sent for sequencing (Macrogen, Korea). Further analyses were carried out using the methods and procedures described above for the isolate sequences. A cluster analysis was constructed with the Dice coefficient, using an unweighted pair-group average with Euclidean distance estimations (GelCompar II software, Applied Maths Biosystems).

### Detection of *Wolbachia* bacteria and *Leishmania* parasite

The intestinal DNA of female pools collected in Ovejas and Colosó was also searched for *Wolbachia* endosymbiont bacteria and the *Leishmania* parasite. The specific primers wsp81F (5'-TGG TCC AAT AAG TGA TGA AGA AAC-3') and wsp691R (5'-AAA AAT TAA ACG CTA CTC CA-3') were used to detect *Wolbachia*. These primers amplify a partial fragment (590–632 bp) of the gene that encodes *Wolbachia* main surface protein (WSP) [48]. The reaction mixture and working conditions used for detecting *Wolbachia* included a 2 μl DNA (30 ng) sample in a final reaction volume of 20 μl [49]. A PCR positive control was included, which consisted of DNA obtained from 10 *Aedes* (*Stegomyia*) *aegypti* larvae (L4) infected with a *Wolbachia* reference strain (Group A, wMel strain) under insectary laboratory conditions.

To detect *Leishmania* infection, the specific primers HSP70-F25 (5'-GGA CGC CGG CAC GAT TKC T-3') and HSP70-R617 (5'-CGA AGA AGT CCG ATA CGA GGG A-3') were used, as described by Fraga et al. [50]. These primers amplify a partial fragment (593 bp) of the HSP-70 N gene (cytoplasmic Heat Shock Protein 70).

### Data analysis

A descriptive analysis was performed to determine possible differences in the gut bacterial load (colony forming units) in adult (fed females, unfed females, males) and immature stages (larvae, pupae) of *Lu. evansi*. The XLSTAT 3.04 (https://www.xlstat.com/es/) program was used to generate a simple correspondence analysis between bacterial isolates and the TTGE profiles, *Lu. evansi* stages and sample origin, in order to distinguish possible trends or associations. Finally, a presence/absence matrix was used to perform an analysis of similarity (ANOSIM) based on the Bray-Curtis index, using PAST software, version 2.17 [51]. The analysis of similarity was used to examine the statistical significance of differences between the TTGE profiles.

## Results

### *Lutzomyia evansi* guts

A total of 752 *Lu. evansi* intestines were dissected and processed form 31 pools, each containing 13–50 adult intestines. Analysis of individual forms was performed for immature stages (Table 1). We obtained taxonomic identifications of adults from both localities by using dichotomous keys [28], and confirmed these with the COI mitochondrial marker. Immature stages were also identified with the COI mitochondrial marker (results not shown).

**Table 1:** Description of samples of adult and immature stages of *Lutzomyia evansi* collected and processed for analysis of bacterial flora associated with the gut

Description	Adult stages						Immature stages		
	Males (M)		Unfed females (UF)		Fed females (FF)		Larvae (L4)	Pupae (PP)	Total
	Colosó	Ovejas	Colosó	Ovejas	Colosó	Ovejas	Ovejas	Ovejas	
Groups analyzed	1	2	7	4	1	2	11	3	31
Number of guts per group	13	36–45	48–50	50	28	18–50	1	1	–
Total number of processed guts	13	81	348	200	28	68	11	3	752
Total number of bacterial isolates obtained^a^	4	2	24	17	3	5	50	20	125

^a^Bacterial isolates evaluated with cluster analysis of the spacer region (ITS) banding pattern between the 23S and 16S ribosomal genes

### Cultured bacterial community of *Lu. evansi*

**Fig. 2:**
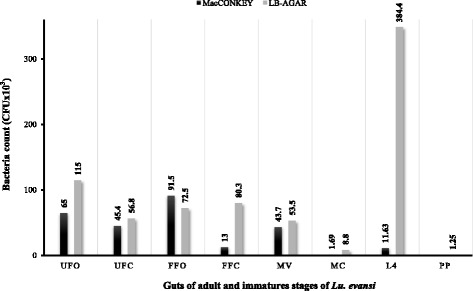
Bacterial counts (CFU) in *Lu. evansi* guts, obtained in media LB agar and MacConkey. *Abbreviations*: FFO, fed females from Ovejas; FFC, fed females from Coloso; UFO, unfed females from Ovejas; UFC, unfed females from Coloso; MC, males from Coloso; MV, males from Ovejas; L4, larvae from Ovejas; PP, pupae from Ovejas

In this study, 125 bacterial strains were isolated from different culture media (LB Agar, MacConkey Agar) (Table 1). The results showed significant differences (Chi-square test: *χ*^2^ = 11.35, *df* = 7, *P* < 0.004) between stages in terms of the CFU count, which was highest for larvae in LB agar (CFU/intestine = 348.4 × 10^-3^), and lowest for pupae in both culture media (CFU /intestine = 1.25 × 10^-3^) (Fig. 2). In adults, the bacterial load was lower in male populations from Colosó (CFU/intestine = 8.8 × 10^-3^) than in the pools of fed females (CFU/intestine = 80.3 × 10^-3^–72.5 × 10^-3^) (Chi-square test: *χ*^2^ = 150.64, *df* = 3, *P* < 0.001) and unfed females (CFU/intestine = 56.8 × 10^-3^–115 × 10^-3^) (Chi-square test: *χ*^2^ = 215.75, *df* = 8, *P* < 0.001) (Fig. 2). After morphological (Gram stain) and molecular characterization of the isolates based on RISA pattern analysis revealed differences of more than 30 %, we found that 72.5 % were Gram-negative and 27.4 % were Gram-positive (mainly pupae). Fifty-one presumably different isolates were selected for 16S rDNA sequencing; 26 isolates from this group were selected for additional *gyr*B gene sequence analysis.

### Identification of bacterial isolates using 16S rDNA sequences

**Fig. 3:**
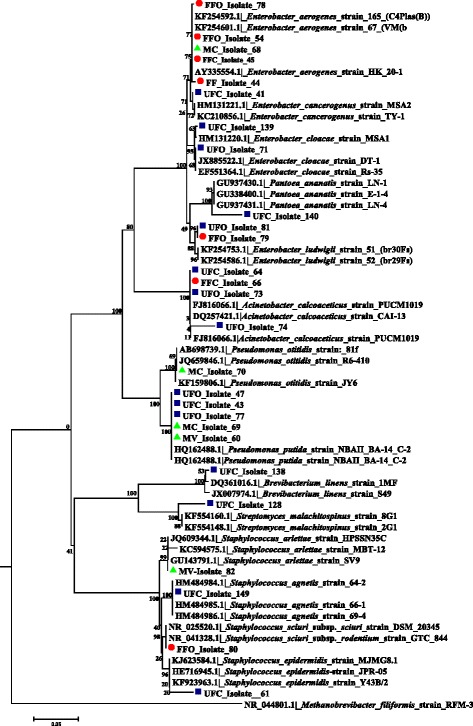
Neighbor-joining dendrogram for partial 16S rDNA sequences of bacteria obtained from adult stages of *Lutzomyia evansi* collected in the municipalities of Ovejas and Colosó (Sucre Department, Colombia), based on the Kimura 2-parameter model. Numbers at the nodes represent bootstrap values. The robustness of the phylogeny was tested by bootstrap analysis using 1000 iterations. *Abbreviations*: FFO, fed females from Ovejas; FFC, fed females from Coloso; UFO, unfed females from Ovejas; UFC, unfed females from Coloso; MC, males from Coloso; MV, males from Ovejas

**Fig. 4:**
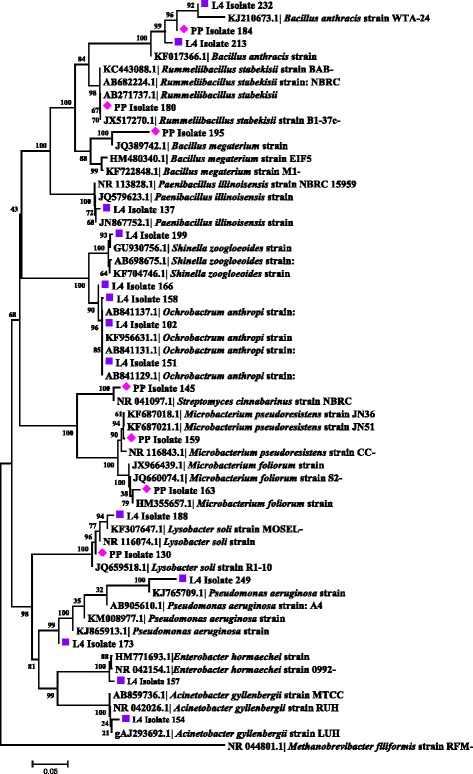
Neighbor-joining dendrogram for partial 16S rDNA sequences of bacteria obtained from immature stages of *Lutzomyia evansi* collected in the Ovejas municipality (Sucre Department, Colombia), based on the Kimura 2-parameter model. Numbers at the nodes represent bootstrap values. The robustness of the phylogeny was tested by bootstrap analysis using 1000 iterations. *Abbreviations*: L4, larvae from Ovejas; PP, pupae from Ovejas

Three different predominant phyla (Proteobacteria, Actinobacteria and Firmicutes) with high similarity percentages (97–100 %) were isolated based on the similarity analysis of the related sequences from RDP and BLAST (NCBI database) (Tables 2 and 3 and Figs. 3 and 4).

**Table 2:** Closest phylogenetic identification of bacteria isolated from the digestive tracts of adult *Lutzomyia evansi*, according to their similarity with 16S rRNA gene sequences in the GenBank and RDP II databases

Group/Locality	Isolate code (GenBank acc. No.)	Related taxa (GenBank acc. No.)	BlastN (%)^a^	Phylum	Found in sand flies, other insect vectors [Reference]	Note [Reference]
Unfed females/COLS	UFC Isolate 61 (KU134756)	*S. epidermidis* (KJ623584.1)	97	Firmicutes	*Cx. quinquefasciatus* [9, 66]	Human and animal skin [74]
	UFC Isolate 149 (KU134739)	*S. agnetis* (HM484986.1)	98	Firmicutes		Human and bovine pathogen [74]
	UFC Isolate 128 (KU134759)	*S. malachitospinus* (KF554160.1)	98	Actinobacteria		Soil, plants, entomopathogen [11]
	UFC Isolate 138 (KU134758)	*B. linens* (DQ361016.1)	98	Actinobacteria	*Ph. argentipes* [53]	Soil, human skin, prebiotic [13]
	UFC Isolate 43 (KU134745)	*P. putida* (HQ162488.1)	99	Proteobacteria	*Lu. longipalpis* [17–19]	Fermentation, plants, genetic potential [13]
	UFC Isolate 64 (KU134748)	*A. calcoaceticus* (FJ816066.1)	98	Proteobacteria	*Cx. quinquefasciatus* [9, 66]	Human pathogen [9]
	UFC Isolate 140 (KU134754)	*P. ananatis* (GU937431.1)	97	Proteobacteria		Plant pathogen, entomopathogen [31]
	UFC Isolate 139 (KU134778)	*E. cloacae* (KC990807.1)	99	Proteobacteria	*Ph. argentipes* [53]; *Lu. longipalpis* [17–19]; *Ph. papatasi* [26, 31]; *An. albimanus* [9]; *An. stephensi* [9]	Human pathogen, antitripanosomic, termites, hydrogen production [72]
	UFC Isolate 41 (KU134743)	*E. cancerogenus* (HM131221.1)	99	Proteobacteria		Human pathogen [20, 52, 57, 65]
	UFC Isolate 42 (KU134744)	Unc. bacterium clone (KF842672.1)	99	–		
	UFC Isolate 63 (KU134752)	Unc. bacterium clone (KF842672.1)	97	–		
Unfed females/OVEJ	UFO Isolate 77 (KU134755)	*P. putida* (HQ162488.1)	99	Proteobacteria	*An. darlingi* [9]; *An. dureni* [9]; *An. funestus* [9]; *An. gambiae* [9]; *An. stephensi* [9]	
	UFO Isolate 47 (KU134740)	*P. putida* (HQ162488.1)	99	Proteobacteria		
	UFO Isolate 74 (KU134753)	*A. calcoaceticus* (FJ816066.1)	95	Proteobacteria		
	UFO Isolate 73 (KU134751)	*A. calcoaceticus* (FJ816066.1)	99	Proteobacteria		
	UFO Isolate 81 (KU134760)	*E. ludwiggii* (KF254586.1)	99	Proteobacteria		
	UFO Isolate 71 (KU134741)	*E. cloacae* (KC990807.1)	98	Proteobacteria		
	UFO Isolate 53 (KU134757)	Unc. bacterium clone (KF842672.1)	97	–		
	UFO Isolate 51 (KU134742)	Unc. bacterium clone (HM557220.1)	98	–		
Fed females/ COLS	FFC Isolate 66 (KU134749)	*A. calcoaceticus* (FJ816066.1)	97	Proteobacteria		
	FFC Isolate 44 (KU134746)	*E. aerogenes* (AY335554.1)	99	Proteobacteria	*Ae. aegypti* [9, 12]¸ *Ph. argentipes* [53]; *Lu. longipalpis* [17–19]; *Ph. papatasi* [26, 31]	*A. mellifera*, simbiont, human pathogen [20, 52, 57, 65]
	FFC Isolate 45 (KU134747)	*E. aerogenes* (AY335554.1)	98	Proteobacteria		
Fed females/ OVEJ	FFO Isolate 80 (KU134762)	*S. sciuri* (NR_025520.1)	99	Firmicutes		Animal skin and mucus [26, 73–75]
	FFO Isolate 79 (KU134799)	*E. ludwiggii* (KF254586.1)	99	Proteobacteria		
	FFO Isolate 54 (KU134761)	*E. aerogenes* (AY335554.1)	99	Proteobacteria		
	FFO Isolate 78 (KU134747.1)	*E. aerogenes* (AY335554.1)	99	Proteobacteria		
Males/ COLS	MC Isolate 69 (KU134763)	*P. putida* (HQ162488.1)	99	Proteobacteria		
	MC Isolate 70 (KU134777)	*P. otitidis* (JQ659846.1)	100	Proteobacteria		Human pathogen (ear infections) [66–70]
	MC Isolate 68 (KU134750)	*E. aerogenes* (AY335554.1)	99	Proteobacteria		
Males/ OVEJ	MV Isolate 82 (KU134762)	*S. arlettae* (GU143791.1|)	99	Firmicutes		
	MV Isolate 60 (KU134764)	*P. putida* (HQ162488.1)	98	Proteobacteria	*Ph. argentipes* [53]	Mammal and bird skin [63, 64]

*Abbreviations*: *COLS* Colosó, *OVEJ* Ovejas, *FFO* fed females from Ovejas, *FFC* fed females from Coloso, *UFO* unfed females from Ovejas, *UFC* unfed females from Coloso, *MC* males from Coloso, *MV* males from Ovejas

^a^Percent similarity

**Table 3:** Closest phylogenetic identification of the 16S rDNA sequences of bacteria isolated from the digestive tracts of immature *Lutzomyia evansi* from the Ovejas locality, according to their similarity with with sequences registered in the GenBank and RDP II databases

Group/Locality	Isolate code (GenBank acc. No.)	Related taxa (GenBank acc. No.)	BlastN (%)^a^	Phylum	Found in sand flies, other insect vectors [Reference]	Note [Reference]
Larvae	L4 Isolate 154 (KU134779)	*P. gyllenbergii* (NR_042026.1)	99	Proteobacteria		
	L4 Isolate 157 (KU134771)	*E. hormaechei* (HM771693.1)	97	Proteobacteria		Human pathogen [20, 52, 57, 65]
	L4 Isolate 173 (KU134783)	*P. aeruginosa* (KJ765709.1)	99	Proteobacteria	*Lu. longipalpis* [17–19]; *Ph. papatasi* [26]; *An. darlingi* [9]; *An. dureni* [9]; *An. funestus* [9]; *An. gambiae* [9]; *An. stephensi* [9]; *Cx. quinquefasciatus* [9, 66]	Human pathogen [16, 71]
	L4 Isolate 249 (KU134786)	*P. aeruginosa* (KJ765709.1)	99	Proteobacteria		
	L4 Isolate 188 (KU134784)	*L. soli* (NR_116074.1)	100	Proteobacteria		Soil, water, plants, entomopathogens, genetic potential [21]
	L4 Isolate 151 (KU134767)	*O. anthropi* (AB841129.1)	99	Proteobacteria		
	L4 Isolate 102 (KU134766)	*O. anthropi* (AB841129.1)	99	Proteobacteria	*Lu. longipalpis* [17–19]	Soil, human pathogen, plants, nematodes, insects and animals, genetic potential [21, 76]
	L4 Isolate 158 (KU134770)	*O. anthropi* (AB841129.1)	99	Proteobacteria		
	L4 Isolate 166 (KU134781)	*O. anthropi* (AB841129.1)	99	Proteobacteria		
	L4 Isolate 199 (KU134768)	*S. zoogloeoides* (GU930756.1)	99	Proteobacteria		Biotechnological and genetic potential, soil [21]
	L4 Isolate 137 (KU134769)	*P. illinoisensis* (JQ579623.1)	98	Firmicutes		Soil, plants, insect larvae, genetic, biotechnological potential, entomopathogenic [21]
	L4 Isolate 213 (KU134765)	*B. anthracis* (KF910781.1)	98	Firmicutes		
	L4 Isolate 232 (KU134785)	*B. anthracis* (KF910781.1)	98	Firmicutes		Entomopathogenic, *A. mellifera*, human, animal pathogen [21]
Pupae	PP Isolate 130 (KU134772)	*L. soli* (NR_116074.1)	98	Proteobacteria		
	PP Isolate 163 (KU134780)	*M. foliorum* (HM355657.1)	98	Actinobacteria		*Musca domestica*, soil, leaves
	PP Isolate 159 (KU134774)	*M. pseudoresistens* (KF687018.1)	99	Actinobacteria		Soil, plants [13]
	PP Isolate 145 (KU134775)	*S. cinnabarinus* (NR_041097.1)	97	Actinobacteria		Soil, entomopathogenic, probiotic [13]
	PP Isolate 195 (KU134776)	*B. megaterium* (JQ389742.1)	97	Firmicutes	*Ph. argentipes* [53]; *Ph. papatasi* [26]; *An. arabiensis* [9]; *An. funestus* [9]; *An. gambiae* [9]; *An. stephensi* [9]	
	PP Isolate 180 (KU134773)	*R. stabekisii* (JX517270.1)	99	Firmicutes		Soil, plants [13]
	PP Isolate 184 (KU134782)	*B. anthracis* (KF910781.1)	100	Firmicutes		

*Abbreviations*: *L4* fourth-instar larvae, *PP* pupae

^a^Percent similarity

The genera *Enterobacter*, *Pseudomonas* and *Acinetobacter* were more frequent in adults (Table 2). The highest number of bacterial genera was found in the larval stage (*n* = 8), with the *Ochrobactrum* spp. being most prominent. The genera *Lysobacter*, *Microbacterium*, *Bacillus*, *Streptomyces* and *Rummeliibacillus* were identified in the pupal stage (Table 3). The diversity of aerobic bacterial flora in *Lu. evansi* from the two localities, urban and wild environments, was represented by 27 phylotypes at the species level (Tables 2 and 3).

Bacterial communities in unfed females from both locations had the species *Acinetobacter calcoaceticus*, *Pseudomona putida* and *E. cloacae* in common (Table 2). For fed females, the species *E. aerogenes* was prominent, and the presence of *A. calcoaceticus* was also observed (Table 2). In males, a slight similarity with the bacterial communities in the fed females was observed, with *P. putida* and *E. aerogenes* present (Table 2). Two species, *Bacillus anthracis* and *Lysobacter soli*, were only found in the guts of *Lu. evansi* larvae and pupae (Table 3). *Ochrobactrum anthropi* appeared more frequently in larvae. The analyses indicated significant variations in the bacterial species, depending on the developmental stage and origin of the insects (Tables 2 and 3).

### Neighbor-joining cluster analysis of 16S rRNA and *gyr*B gene sequences from bacterial isolates

Molecular identification using 16S rDNA sequences from *Lu. evansi* isolates showed a high level of correspondence with sequences deposited in BlastN at species level and among different taxonomic divisions (Figs. 3 and 4). The analysis showed that most clusters had bootstrap values ranging between 96 and 100 % for bacterial isolates from *Lu. evansi* guts from different stages, geographical locations and types of natural environment (wild and urban). This was observed in the *P. putida* and *A. calcoaceticus* clusters in adults (Fig. 3) and in the *L. soli* and *B. anthracis* clusters in immature specimens (Fig. 4).

**Fig. 5:**
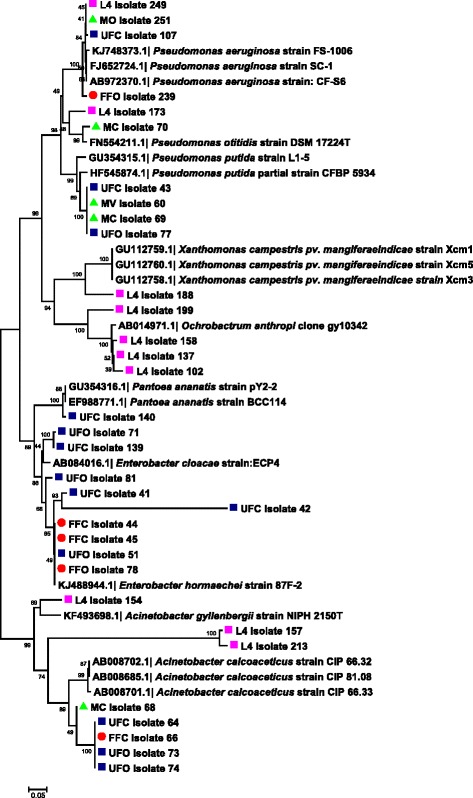
Neighbor-joining dendrogram for *gyr*B gene sequences of bacteria obtained from adult and immature stages of *Lutzomyia evansi* collected in the municipalities of Ovejas and Colosó (Sucre Department, Colombia), based on the Kimura 2-parameter model. Numbers in nodes represent bootstrap values. The robustness of the phylogeny was tested by bootstrap analysis using 1000 iterations. *Abbreviations*: FFO, fed females from Ovejas; FFC, fed females from Coloso; UFO, unfed females from Ovejas; UFC, unfed females from Coloso; MC, males from Coloso; MV, males from Ovejas; L4, larvae from Ovejas

For some isolates, the identity was confirmed with the *gyr*B gene sequences due to difficulties that have been reported with some genera, including *Enterebacter* and *Pseudomonas*, particularly for environmental samples [38]. In our study, the *gyr*B molecular marker worked correctly with *Pseudomonas* species and other genera. This result is consistent with the results of the neighbor-joining analysis of the *gyr*B gene sequence, showing clusters and similar bootstrap values (99–100 %) for most of the bacterial isolates (*A. calcoaceticus*, *Pantoea ananatis*, *O. anthropi*, *P. putida*, *P. aeruginosa* and *P. otitidis*) that were also identified with the 16S rRNA gene sequence (Fig. 5).

### PCR-TTGE and phylogenetic analysis of *L. evansi* intestinal bacteria

**Fig. 6:**
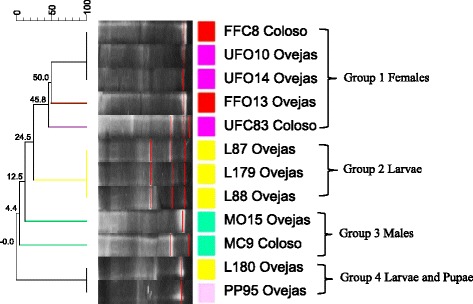
Dice-UPGMA clustering analysis of bacterial diversity obtained of PCR-TTGE Profiles of 16S rDNA amplification products (V3–V6 variable regions), through GelCompar II software. *Abbreviations*: L, larvae; MO, males from Ovejas; MC, males from Coloso; FFO, fed females from Ovejas; FFC, fed females from Coloso; UFO, unfed females from Ovejas; UFC, unfed females from Coloso. Numbers at the nodes represent the percent similarity between the profiles of banding in the samples analyzed, based on migration with respect to temperature melting of ribosomal amplicons, as well the intensity and thickness of the band that symbolizes the dominance of the microbial community

**Fig. 7:**
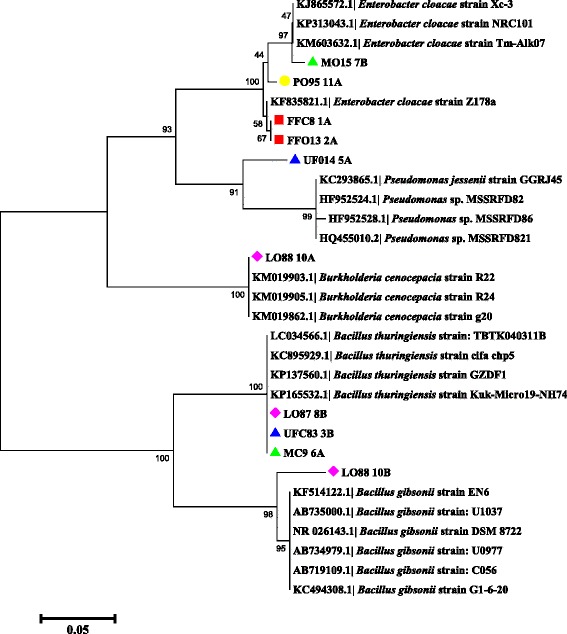
Neighbor-joining dendrogram of 16S rDNA fragment sequences (~450 bp) obtained from the PCR-TGGE profiles, based on the Kimura 2-parameter model. Numbers in nodes represent bootstrap values. The robustness of the phylogeny was tested by bootstrap analysis using 1000 iterations. FFO, fed females from Ovejas; FFC, fed females from Coloso; UFO, unfed females from Ovejas; UFC, unfed females from Coloso; MC, males from Coloso; MV, males from Ovejas; LO, larvae from Ovejas; PO, pupae from Ovejas

TTGE band profiles and analysis of partial 16S rDNA sequences indicative of different genera associated to adult and immature stages of *Lu. evansi* were compared with 16S rDNA sequence information for known bacteria retrieved from the GenBank database. The banding patterns were similar among fed females in both geographical locations, but indicated significant variations between the bacterial communities in unfed females and males (Fig. 6). The predominant TTGE bands for *L. evansi* intestinal bacteria indicated similarity values between 99 and 100 %, corresponding to *E. cloacae* and *B. thuringiensis* (Table 4), in adult and immature specimens (larvae and pupae). An uncultured bacterium sequence was detected only in samples from the Colosó region (Table 4). All the TTGE sequences obtained in this study have been registered in GenBank (Table 4). In the case of the larvae, we found a different banding pattern than that expressed in adults and pupae (Fig. 6). This pattern was related to the *Burkholderia cenocepacia* and *Bacillus gibsonii* bands. Partial 16S rDNA sequences of the bands were identified through comparison with the sequences available in the Genbank database and RDP II, with high similarity percentages found (97–100 %) (Table 4). The NJ topology generated comprised clusters with suitable bootstrap support (98–100 %), indicating that the phylogenetic affiliations were consistent (Fig. 7).

**Table 4:** Closest phylogenetic classification of bacteria found in the digestive tracts of adult and immature *Lutzomyia evansi* by TTGE analysis, according to their similarity with 16S rRNA gene sequences recorded in the GenBank and RDP II databases

Group	TGGE band code (GenBank acc. No.)	Locality	Related taxa (GenBank acc. No.)	BlastN (%)^a^	Phylum
Larvae	L87-8B (KU134787)	Ovejas	*B. thurigiensis* (KP137560.1)	99	Proteobacteria
	L88-10A (KU134788)	Ovejas	*B. cenocepacia* (KM019903.1)	100	Proteobacteria
	L88-10B (KU134789)	Ovejas	*B. gibsonii* (KF514122.1)	99	Firmicutes
	L180-11 (KU134790)	Ovejas	*E. cloacae* (KF429490.1)	99	Proteobacteria
Fed females	FFC8-1A (KU134791)	Coloso	*E. cloacae* (KF429490.1)	100	Proteobacteria
	FFO13 2A (KU134792)	Ovejas	*E. cloacae* (KF429490.1)	99	Proteobacteria
Unfed females	UFC83-3A (KU134793)	Coloso	*Pseudomonas* sp. (HF952524.1)	89	Proteobacteria
	UFC83-3B (KU134794)	Coloso	*B. thurigiensis* (KP137560.1)	99	Firmicutes
	UFC83-3C (KU134795)	Coloso	Uncultured bacterium clone (KF973118.1)	99	
	UF014-5A (KU134796)	Ovejas	*Pseudomonas* sp. (KC293865.1)	89	Proteobacteria
Males	MC9-6A (KU134797)	Coloso	*B. thurigiensis* (KP137560.1)	99	Proteobacteria
	MC9-6B (KU134798)	Coloso	Uncultured bacterium clone (KF973118.1)	99	
	MV15-7B (KU134799)	Ovejas	*E. cloacae* (KF429490.1)	99	Proteobacteria

^a^ Percent similarity

### Bacterial diversity of *Lu. evansi* guts associated with blood intake and developmental stage

**Fig. 8:**
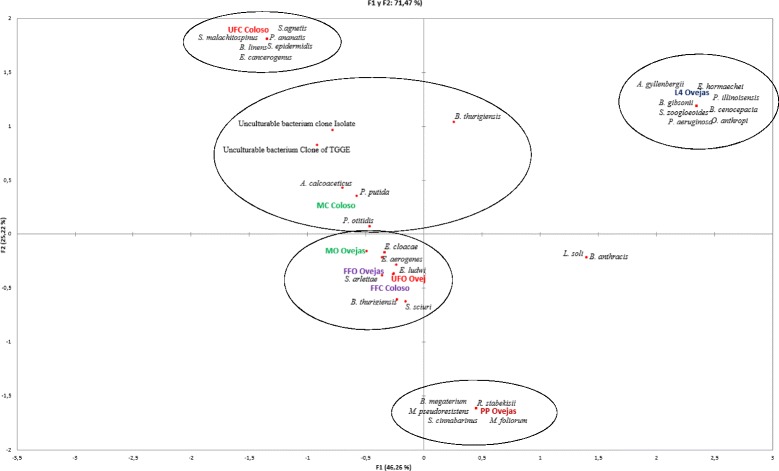
Simple correspondence analysis of the intestinal bacterial flora identified with partial 16S rDNA sequences of isolates and fragment sequences (~450 bp) obtained from the PCR-TGGE profiles. *Abbreviations*: UFO, unfed females from Ovejas; UFC, unfed females from Coloso; FFO, fed females from Ovejas; FFC, fed females from Coloso; MC, males from Coloso; MO, males from Ovejas; PP, pupae; L4, larvae. The XLSTAT 3.04 (https://www.xlstat.com/es/) program was used to generate a simple correspondence analysis. The dots symbolize the location of the group of bacteria (consortia) associated with intestines of *Lu. evansi*, using ellipses of black color

In order to statistically analyze the TTGE bacterial profiles relative to the developmental and nutritional status of the insects, banding patterns were analyzed with Gel Compare II software. These revealed the formation of four groups of clearly differentiated bacterial communities (Fig. 6). Nevertheless, statistically significant differences were found between the banding patterns associated with the bacterial communities present in the guts of unfed females from different locations (one-way ANOSIM test: *R* = 0.8674, *P* = 0.0001); fed females from Colosó and unfed females from Ovejas (one-way ANOSIM test: *R* = 0.7651; *P* = 0.0001); and males from both locations (one-way ANOSIM test *R* = 0.8821, *P* = 0.0001). In immature specimens, there were no statistically significant variations among guts of larvae, but there were variations between the larvae and adult stages (one-way ANOSIM test: *R* = 0.8345, *P* = 0.001). This indicates that bacterial communities obtained by independent methods of cultivation, present variation depending on the origin and stages of *Lu. evansi*. A simple correspondence analysis explaining 71.47 % of the variation in the dataset and integrating the results obtained by both approaches (TGGE of the total fraction and the cultivable fraction), revealed various bacterial consortia specifically associated with the guts of larvae, pupae and unfed females (Colosó population) (Fig. 6). In contrast, the groups of males, fed females and unfed females (Ovejas population) analyzed, shared a bacterial species consortium, indicating greater proximity between the microbiota of males and fed females (Fig. 8). This suggests the complementary contribution of both approaches to the study of communities of bacteria.

### Bacterial diversity of *Lu. evansi* guts associated with the geographical origin of *Lu. evansi*

In general, the natural populations of *Lu. evansi* had the following bacterial genera in common: *Pseudomonas*, *Acinetobacter*, *Enterobacter*, *Staphylococcus* and *Streptomyces*. However, some differences are described according to the geographical provenance. In the jungle environment, the microbiota associated with the gut of *Lutzomyia evansi*, contained bacterial genera (*Brevibacterium* and *Pantoea*) that were not present in the urban environment. Other bacterial genera such as *Lysobacter*, *Ochrobactrum*, *Shinella*, *Paenibacillus*, *Bacillus*, *Microbacterium* and *Rummeliibacillus*, could be distinguished associated with substrates extracted from potential breeding sites (base of *Cordia dentata* shrubs) and from the urban environment of Ovejas. In conclusion, there is a greater number of bacterial communities associated with urban populations of *Lu. evansi*.

### Detection of *Wolbachia* and *Leishmania* in guts of female *Lu. evansi*

Given that *Wolbachia* is very important in the identification of potential strains with the ability to interrupt the life-cycle of *Lu. evansi* or to block the development of parasites or viruses, and *Leishmania* is the most relevant parasite to control in *Lu evansi*, their presence in our insect samples was examined by PCR. *Wolbachia* and *Leishmania* were not detected or found to be amplified in the intestinal groups formed by female *Lu. evansi* in our conditions.

## Discussion

To our knowledge, this study represents the first report of bacteria associated with the guts of natural populations of adult and immature *Lu. evansi* present in both types of environment (urban and jungle biotypes) on the Caribbean coast of Colombia, based on a polyphasic approach including culture-dependent and culture-independent methods. The analyses permitted identification of bacterial species, with a percentage of microorganisms or cultivable phylotypes (3–5 %) obtained by the culture-dependent method under aerobic conditions [17–20]. For this reason, and to increase the range of bacteria, we examined the microbial diversity associated with *Lu. evansi* guts in various development stages, using the PCR-TTGE technique and DNA sequence analysis [17–20, 26, 51–53]. We found significant variations in the bacterial communities associated with *Lu. evansi* guts, depending on the developmental stage (adult and immature stages), the food source (female blood intake) and location.

Many studies on intestinal microbiota do not include immature stages of sand flies because of the difficulties in finding at the natural breeding sites, the complexity of transporting them alive to the laboratory to obtain the guts, and the lack of taxonomic keys to identify them to species level [21, 54]. In this sense, it is necessary to highlight the usefulness of the COI gene barcode sequences, which enabled the taxonomic association of immature and adult specimens of *Lu. evansi* (data not shown) in a manner that was consistent with other studies in terms of intraspecific divergence percentages (0.002 to 0.031) and neighbor-joining cluster supports (98–100 %) [55, 56].

This approach provided information on unregistered bacterial communities belonging exclusively to these stages [21, 22, 53, 54]. The majority of bacterial species in immature specimens were Gram-positive and were associated with the soil, which is directly related to their habitat. However, most of these were transient bacteria and none were detected in the adult stages, presumably as a result of intestinal resorption [57–59]. Bacterial load obtained by dependent cultivation methods was similar to that reported for adults states of sand flies, while the bacterial load of larvae was significantly higher and this can be justified by the wide range of bacteria that can exist in the soil or natural breeding sites [18, 20]. This indicates that the platform used to access the cultivable intestinal microbiota is adequate and reproducible, because of the high number of isolates that were identified later.

A wide representation of Gram-negative bacteria was detected in females, males and larvae belonging to the genera *Pseudomonas*, *Acinetobacter*, *Enterobacter* and *Ochrobactrum*, while in the pupal stages all bacteria were Gram-positive and included the genera *Microbacterium*, *Streptomyces*, *Bacillus*, *Lysobacter* and *Rummeliibacillus*. Studies of gut bacteria from wild and laboratory populations of *Lu. longipalpis*, *Phlebotomus* and different vector insects have shown a larger proportion of Gram-negative bacteria [19, 60–62]. The dominance of Gram-negative bacteria may be associated with partial or complete inhibitory activity on the development of parasites (*Plasmodium falciparum*, *Leishmania infantum chagasi*) and the multiplication of viruses (Dengue) in the guts of some mosquito species [31].

Fewer Gram-positive bacteria were found in the gut of *Lu. evansi*. The mechanisms of action of the toxins produced by Gram-positive bacteria associated with insect guts are related to alkaline pH, and the responses from the insects’ immune systems are varied [15, 60–62]. These bacteria are characterized by their ability to adapt to different environmental niches and insect intestinal systems [17], which could explain their association with immature stages (Table 3).

We suggest that differences in the microbiota associated with the gut of *Lu. evansi* may be the result of the fauna and flora in the two collection sites, as also of the influence of soil composition in Ovejas [17, 20, 57]. The anthropophilic habit, the need to ingest blood (blood-sucking habit) and get various sources of carbohydrates (phytophage habit), allows *Lu. evansi* to obtain a variety of bacteria from the skin of humans and animals as well as from plants. In unfed *Lu. evansi* females from both locations, the main species identified were *A. calcoaceticus*, *P. putida* and *E. cloacae. Acinetobacter calcoaceticus* is considered a human pathogen [63, 64], and although the presence of this specific species has not been previously reported in sand flies, studies on mosquitoes (*Anopheles*, *Aedes* and *Culex*) and sand flies (*P. argentipes* and *Lu. longipalpis*) have detected the presence of species of the genus *Acinetobacter* [20, 53, 65, 66]. This suggests that the genus *Acinetobacter* is dominant in vector insects and is considered a “core genus” in the guts of natural *Anopheles* populations [65].

*Pseudomona putida* isolates were discovered both in the guts of unfed *Lu. evansi* females and in males. Similar results were found in the guts of a natural population of *Lu. longipalpis* collected from a visceral leishmaniasis-endemic area in Brazil (Jacobina, State of Bahia) [20]. *Pseudomona putida* is associated with fermented foods and plants, has good potential for genetic transformation, and is contained within the group of non-pathogenic bacteria that easily adapt to the physiological and immunological conditions present in the mosquito midgut [18]. The fact that *P. putida* was present in both locations and in different stages (unfed females and males) could be associated with the food source [18].

Regarding the presence of the genus *Pseudomonas* in *Lu. evansi* guts, it is necessary to highlight the presence of *P. aeruginosa* and *P. otitidis* isolates in the guts of male and larval populations collected in Colosó and Ovejas, respectively. These two species are opportunistic human pathogens that appear in immunocompromised patients, and their presence have been recorded in other insects (Table 2) [20, 65–70]. *Pseudomona aeruginosa* have the capacity to synthesize antiparasitic molecules (prodiogiosine, cytotoxic metalloproteinase, haemolysins, antibiotics and hemagglutinin) in insect guts [16, 71].

The discovery of *E. cloacae* isolates in the guts of unfed *Lu. evansi* females is consistent with previously recorded findings in sand flies and mosquitoes (*Ph. argentipes*, *Lu. longipalpis*, *Ph. papatasi*, *Ph. sergenti*, *An. albimanus* and *An. stephensi*) [31, 53, 72]. Gouveia et al. [20] suggested that *E. cloacae* does not influence parasite establishment in sand fly guts in leishmaniasis areas. However, other studies have shown that *Enterobacter* spp. (*Enterobacter* sp. strain *Eng Z*) can make insects more resistant to infections from other bacteria and can partially or fully inhibit ooquinete, sporozoite and oocyst formation in *Anopheles* [71, 72]. Other species of *Enterobacter* associated with *Lu. evansi* guts and considered pathogenic, including *E. cancerogenus*, *E. ludwiggii*, *E. aerogenes* and *E. hormaechei*, were detected in other insect vectors (Table 2) [20, 52, 57, 65].

There was a strong correlation between *Lu. evansi* gut bacteria and the potential source of bacterial infection, which is associated with the behavior and/or eating habits of this species (Fig. 6). The presence of *B. linens*, *S. epidermidis*, *S. sciuri* and *S. agnetis* is associated with the guts of fed and unfed females. These bacteria are important because of their close relationship with the skin and mucosa in both humans and animals [26, 73–75], which constitute the main source of blood intake for *Lu. evansi* females, due to their anthropophilic and/or zoophilic characteristics [5].

A wide variety of bacteria with entomopathogenic or genetic potential were isolated mainly in the guts of *Lu. evansi* larvae and pupae (*O. anthropi*, *L. soli*, *P. illinoisensi*, *B. anthracis* and *S. zoogloeoides*, among others) collected from natural breeding sites in the Ovejas municipality (Table 3). Previous studies have indicated that these bacteria are associated with plant roots, decaying plant material, straw and forage, which are components of natural microhabitats where immature sand flies develop [21]. Recent studies suggest that *Ochrobactrum* species reduce *Leishmania* establishment in the gut and negatively impact *Lu. longipalpis* survival [76].

The study of microbiota for paratransgenic insect generation also involves the detection and isolation of symbionts like *Wolbachia*, and the determination of their influence on the vector insect’s life-cycle and on parasite or virus obstruction [77]. The culture-dependent and culture-independent approaches we used failed to identify these symbionts, possibly because these intracellular species have little representation in the gut and are difficult to isolate in conventional culture media [78]. However, it is important to emphasize the detection of the symbiont *Pa. ananatis*, which is related to plants and soils and is classified as entomopathogenic [31].

The culture-independent approach permitted differentiation of the structure of gut bacterial communities in developmental stages of natural populations of *Lu. evansi*. The most important results include the discovery of variations depending on the nutritional status of females and the wide variety of bacteria associated with immature stages. This technique has only been previously evaluated for the analysis of bacterial communities in *P. dubosqui* and *P. papatasi* sand flies [26]; the findings indicated low bacterial community diversity dominated by *Microbacterium* in wild adult stages and Chloroflexi in laboratory-reared immature stages [26].

The presence and abundance of *E. cloacae* was noteworthy mainly in males and females from both populations and in pupae from Colosó. These results suggest that *E. cloacae* is a resident population associated with *Lu. evansi*, with possible transstadial transmission. Other bacteria identified through the TTGE technique include *Burkholderia cenocepacia*, *Bacillus thuringiensis*, *Bacillus gibsonii* and uncultured bacteria, which were not detected by the culture-dependent approach.

*Burkholderia cenocepacia* was found only in *Lu. evansi* larvae from the Ovejas locality; this bacterial species belongs to the symbiont group with high genetic diversity (strains) associated with insects. Depending on the strain, it may have adverse physiological functions (growth retardation, mortality of nymphs or immature stages and/or sterility), positive physiological functions (fitness or protection of the insect against entomopathogenic fungi), or metabolic functions (nitrogen fixation ability) [79, 80]. Members of *Burkholderia* are strictly associated with soil and are commonly found in plant roots, adjacent areas and other moist environments. This explains the presence of *Bu. cenocepacia* in *Lu. evansi* larvae, which develop in this type of substrate [79].

The soil bacteria *B. thuringiensis* and *B. gibsonii* are of great importance in the biocontrol of agricultural pests and biotechnological applications [81], and can greatly affect the intestinal bacterial community of the host insect [81, 82]. These two species have not been reported among bacterial flora associated with sand fly guts.

Studies of gut microbiota in sand flies have mainly used the 16S molecular marker via direct sequencing of amplicons from isolates differentiated by morphology, cloned amplicons from total DNA, TTGE/DGGE techniques, and next-generation sequencing strategies [17, 19, 26, 31, 57]. However, the occurrence of multiple copies of the 16S gene in some bacteria and the sequence heterogeneity between these copies may complicate ecological interpretation by causing confusion about the taxonomic identity of closely related species, as in the genera *Enterobacter* and *Pseudomonas* [83]. Furthermore, it has been shown that 16S gene heterogeneity is typical in bacteria isolated from natural environments [84].

The *gyr*B gene, on the other hand, exists as a single copy in bacterial genomes thus allowing a more accurate differentiation between species and/or populations, with some taxonomic conflicts reported in genera such as *Pseudomonas*, *Bacillus* and Enterobacteriaceae, lactic acid bacteria and mycobacteria [85]. However, the results were not as good for other species. Low bootstrap values (89 %) were found for the cluster related to *E. hormaechei* and *E. cloace*, and no taxonomic definition was found for the isolates L4_Isolate 199 and L4_Isolate 188, which were identified by analysis of the16S rDNA gene as *Shinella zoogloeoides* and *L. soli*. This result may be attributed to the absence of *gyr*B sequences in GenBank for these species; however, there is some relationship with *Xanthomonas campestris*, a phylogenetically related species.

We indicate that due to the environmental plasticity of *Lu. evansi*, cultivation-based techniques have been unable to accurately capture the true diversity within microbial communities [86]. To address the lack of information for species that are recalcitrant to cultivation, the recent use of “*culture*-*independent methods*” based on DNA sequencing technology advances (next-generation sequencing-NGS) have established distinctive solid profiles of microbial communities from different samples [26]. Further studies would be needed to determine whether the observed changes in bacterial communities associated with the different developmental stages and blood sources of the insects could influence the development and transmission of the *Leishmania* parasite, using Illumina-based 16S rRNA gene sequencing. This could be achieved by utilizing next-generation sequencing methods for an in-depth comparative analysis of the different feeding stages and/or parasitic infection in *Lu. evansi*.

In this study, *Leishmania* was not detected. Other researchers have reported differences in the sensitivity of different molecular markers and conventional tests (PCR, RFLP, isozyme patterns, hybridization with DNA probes) for the detection, diagnosis and identification of *Leishmania* species; and they propose that exploring the possibility of viewing promastigotes by the dissection of digestive tracts and the implementation of more variants of PCR with genus-specific primers would be beneficial [6, 50]. However the prevalence of natural infections with *Leishmania* in sand flies is low and the process of simultaneous identification of *Leishmania* and gut microbiota can be complicated.

Finally, further studies may include the study of bacterial communities under anaerobic conditions; there is a possibility of finding a higher diversity. Oxygenation of insect guts varies from fully aerobic to anaerobic, with anaerobic conditions more common in insects that have enlarged gut compartments and robust gut communities, such as sand flies [87].

## Conclusions

This study may serve as a starting point for understanding particular aspects of the interaction and dynamic changes in gut bacterial communities in the developmental stages of *Lu. evansi* from Colombia’s Caribbean coast. TTGE profiles and culture-dependent methods are efficient and complementary. These methods work together to record and increase the range of bacterial communities detected in *Lu. evansi* guts. The dominant bacterial communities associated with *Lu. evansi* belong to the genera *Enterobacter*, *Pseudomonas*, *Acinetobacter* and *Ochrobactrum*. Additionally, the large number of bacterial species reported may have biological and genetic potential for anti-tripanosomal or biological control assays in leishmaniasis vectors.

## Abbreviations

COI: Cytochrome *c* oxidase subunit I gene
TTGE: Temporal temperature gradient gel electrophoresis
*gyrB*: Gene that encodes the subunit B of DNA gyrase, a type II DNA topoisomerase
COLS: Colosó
OVEJ: Ovejas
FFO: Fed females from Ovejas
FFC: Fed females from Coloso
UFO: Unfed Females from Ovejas
UFC: Unfed Females from Coloso
MC: Males from Coloso
L4: Fourth-instar larvae
PP: Pupae
